# MEG Beamformer-Based Reconstructions of Functional Networks in Mild Cognitive Impairment

**DOI:** 10.3389/fnagi.2017.00107

**Published:** 2017-04-25

**Authors:** Maria E. López, Marjolein M. A. Engels, Elisabeth C. W. van Straaten, Ricardo Bajo, María L. Delgado, Philip Scheltens, Arjan Hillebrand, Cornelis J. Stam, Fernando Maestú

**Affiliations:** ^1^Laboratory of Neuropsychology, Universitat de les Illes BalearsPalma de Mallorca, Spain; ^2^Networking Research Center on Bioengineering, Biomaterials and NanomedicineMadrid, Spain; ^3^Alzheimer Center and Department of Neurology, Neuroscience Campus Amsterdam, VU University Medical CenterAmsterdam, Netherlands; ^4^Department of Clinical Neurophysiology and MEG Center, Neuroscience Campus Amsterdam, VU University Medical CenterAmsterdam, Netherlands; ^5^Nutricia Advanced Medical Nutrition, Nutricia ResearchUtrecht, Netherlands; ^6^Laboratory of Cognitive and Computational Neuroscience, Center for Biomedical Technology, Complutense University of Madrid and Technical University of MadridMadrid, Spain; ^7^Seniors Center of the District of ChamartínMadrid, Spain; ^8^Department of Basic Psychology II, Complutense University of MadridMadrid, Spain

**Keywords:** mild cognitive impairment, magnetoencephalography, phase lag index, brain networks, minimum spanning tree

## Abstract

Subjects with mild cognitive impairment (MCI) have an increased risk of developing Alzheimer’s disease (AD), and their functional brain networks are presumably already altered. To test this hypothesis, we compared magnetoencephalography (MEG) eyes-closed resting-state recordings from 29 MCI subjects and 29 healthy elderly subjects in the present exploratory study. Functional connectivity in different frequency bands was assessed with the phase lag index (PLI) in source space. Normalized weighted clustering coefficient (normalized Cw) and path length (normalized Lw), as well as network measures derived from the minimum spanning tree [MST; i.e., betweenness centrality (BC) and node degree], were calculated. First, we found altered PLI values in the lower and upper alpha bands in MCI patients compared to controls. Thereafter, we explored network differences in these frequency bands. Normalized Cw and Lw did not differ between the groups, whereas BC and node degree of the MST differed, although these differences did not survive correction for multiple testing using the False Discovery Rate (FDR). As an exploratory study, we may conclude that: (1) the increases and decreases observed in PLI values in lower and upper alpha bands in MCI patients may be interpreted as a dual pattern of disconnection and aberrant functioning; (2) network measures are in line with connectivity findings, indicating a lower efficiency of the brain networks in MCI patients; (3) the MST centrality measures are more sensitive to detect subtle differences in the functional brain networks in MCI than traditional graph theoretical metrics.

## Introduction

### Mild Cognitive Impairment

Mild cognitive impairment (MCI) is an intermediate state between normal aging and dementia. Patients suffering from MCI have an increased risk of developing dementia, in particular Alzheimer’s disease (AD; Shah et al., 2000; Farias et al., 2005). Those MCI subjects with positive biomarkers for AD (i.e., amyloid deposition and neural injury markers such as accumulations of intracellular tau or medial temporal lobe atrophy [MTA]) are regarded to be at the symptomatic pre-dementia phase of AD, and are often referred to as “MCI due to AD” (Albert et al., 2011). The pathological processes that these biomarkers indicate are well described in AD (Braak and Braak, 1991) and are known to produce synaptic disruptions. AD has been described as a “disconnection syndrome,” not only at the cellular level, but also at the macroscale, since the connections in the brain networks also seem to be disrupted (Blennow et al., 1996; Selkoe, 2002; Delbeuck et al., 2003; Arendt, 2009; Takahashi et al., 2010). In fact, structural and functional changes have been described in MCI subjects, suggesting that this disconnection of brain networks already begins during the MCI-stage of AD (Pijnenburg et al., 2004; Koenig et al., 2005; Buldú et al., 2011; Wang et al., 2013).

### Functional Connectivity

One of the main concepts used to understand how the different brain areas interact is functional connectivity (Friston, 1994), which reflects the statistical interdependencies between two-time series of physiological activity. Several resting-state electroencephalography (EEG) and magnetoencephalography (MEG) studies have found decreases in functional connectivity, especially in higher frequency bands (i.e., alpha and beta bands), in MCI patients compared to healthy controls (Moretti et al., 2008; Gómez et al., 2009; López et al., 2014b; Cuesta et al., 2015). This change in synchronization pattern is quite similar to that described for AD patients (Stam and van Dijk, 2002; Jeong, 2004; Stam et al., 2006), although increased connectivity has also been described for AD involving posterior brain regions (Stam et al., 2006; Alonso et al., 2011). Reductions in functional connectivity in MCI have also been observed between regions of the default mode network (DMN), with a parallel disruption of the anatomical connections (Garcés et al., 2014; Pineda-Pardo et al., 2014). However, some resting-state studies comparing different MCI groups have also detected a specific hypersynchronization pattern in high frequency bands (alpha and beta) in those MCI subjects who finally developed AD (López et al., 2014a), or those who presented abnormal concentration of phospho-tau (p-tau) protein in the cerebrospinal fluid (CSF; Canuet et al., 2015). In a recent multicenter study, this profile of hypersynchronization was used to obtain a high percentage of correct classification of MCI and healthy controls (Maestú et al., 2015).

### Brain Networks

Based on the estimated functional connectivity between time series, a weighted network can be reconstructed using graph theory (Bullmore and Sporns, 2009). For this purpose, brain systems are described as sets of nodes (i.e., brain regions or sensors) and links (i.e., functional connections between nodes). The topology of these networks can then be characterized, for example, providing information about the local integration of the network (e.g., the “clustering coefficient”) as well as the global integration (e.g., the “path length”; see Rubinov and Sporns, 2010 for a review). A small-world network has a high local connectedness (quantified by a large clustering coefficient) and a high global integration (quantified by a short path length) and has been regarded as a network with an optimal topology for the transfer of information. AD patients exhibit brain networks that appear to have a sub-optimal topology in which the networks have shifted toward a more random configuration. This was mainly characterized by a loss of small-worldness (see Tijms et al., 2013 for a review), supporting the hypothesis of the disconnection syndrome. However, Tijms et al. (2013) also show that the results differ drastically between studies. Only few studies, using different approaches and modalities, have explored the network topology in MCI, reporting a disturbed balance between local and global integration [functional Magnetic Resonance imaging (fMRI); Wang et al., 2013; MEG; Buldú et al., 2011; Pineda-Pardo et al., 2014]. Methodological difficulties make the comparison between networks of different sizes and different edge densities challenging, if not impossible (Lee et al., 2006; van Wijk et al., 2010; Stam, 2014; Tewarie et al., 2015). This might lead to contradicting results that could be due to differences in modalities (Tijms et al., 2013), but also due to methodological biases (van Wijk et al., 2010). One solution is to reconstruct the minimum spanning tree (MST; Stam, 2014). The MST is a sub-graph of the complete network, which forms a backbone of the original graph. It is uniquely defined whilst avoiding the arbitrary choices of traditional approaches, therefore solving the limitations of previous graph studies (van Wijk et al., 2010; Tewarie et al., 2015). Despite its advantages and application in other neurological disorders (Ortega et al., 2008; Fraschini et al., 2014; Olde Dubbelink et al., 2014; Otte et al., 2015; Tewarie et al., 2015), there is only one fMRI study and one EEG study that has used the MST in comparing healthy elders and AD patients (Çiftçi, 2011; Engels et al., 2015), and none that studied the MST in patients with MCI.

For this reason, we performed an MEG study with MCIs and healthy controls with the aim to characterize how the functional network organization in the MCI stage differs from that of controls. To this end, we estimated MEG resting-state functional connectivity between cortical regions and characterized the topology of the reconstructed MST. We expected to find subtle differences in functional connectivity between MCI patients and controls while the graph theoretical measures will show a clear disrupted topological pattern in MCI. We expected that the novel MST measures give more insight in the network changes of MCI than the traditional network measures (normalized Cw and normalized Lw).

## Materials and Methods

### Subjects

Magnetoencephalography recordings were obtained from 58 subjects (29 MCI patients and 29 healthy elderly subjects). The MCI group was recruited from the Hospital Universitario San Carlos (Madrid), and the control group from the Seniors Center of the District of Chamartin (Madrid). All subjects were right handed (Oldfield, 1971) and native Spanish speakers.

All participants were screened by means of standardized diagnostic instruments and also received an exhaustive neuropsychological assessment. To evaluate their global and cognitive functional status there were used: the Spanish version of the Mini-Mental State Examination (MMSE; Lobo et al., 1979), the Global Deterioration Scale (GDS; Reisberg et al., 1982), the Functional Assessment Questionnaire (FAQ; Pfeffer et al., 1982), the Geriatric Depression Scale-Short Form (GDS-SF; Yesavage et al., 1982), the Hachinski Ischemic Score (Rosen et al., 1980), the questionnaire for Instrumental Activities of Daily Living (Lawton and Brody, 1969), and the Functional Assessment Staging (FAST; Auer and Reisberg, 1997).

Additionally, all subjects underwent an extensive neuropsychological assessment to explore their cognitive functioning by using the following tests: direct and inverse digit span test (DDS and IDS, Wechsler Memory Scale III, WMS-III; Wechsler, 1997), immediate and delayed recall (IR and DR, WMS-III; Wechsler, 1997), phonemic and semantic fluency (PhF and SF, controlled oral word association test; Benton and Hamsher, 1989), ideomotor praxis of Barcelona test (IP; Peña-Casanova, 1990), Visual Object and Space Perception Test (VOSP; Warrington and James, 1991), Boston Naming Test (BNT; Kaplan et al., 1983), and Trail-Making Test (TMT), parts A and B (TMT-A and TMT-B; Reitan, 1958).

The MCI diagnosis was established according to the National Institute on Aging- Alzheimer Association (NIA-AA) criteria (Albert et al., 2011), which includes: (i) self- or informant-reported cognitive complaints; (ii) objective evidence of impairment in one or more cognitive domains; (iii) preserved independence in functional abilities; and (iv) not demented (McKhann et al., 2011). Besides meeting the clinical criteria, MCI participants had signs of neuronal injury (hippocampal volume measured by magnetic resonance imaging (MRI). So, they might be considered as “MCI due to AD” with an intermediate likelihood (Albert et al., 2011).

None of the participants had a history of psychiatric or neurological disorders (other than MCI). General inclusion criteria were: an age between 65 and 80, a modified Hachinski score ≤ 4, a short-form Geriatric Depression Scale score ≤ 5, and T1/T2-weighted MRI within 12 months and 2 weeks before MEG screening without indication of infection, infarction, or focal lesions (rated by two independent experienced radiologists; Bai et al., 2012). Patients were off those medications that could affect MEG activity, such as cholinesterase inhibitors, 48 h before recordings.

### Ethics Statement

Methods were carried out in accordance with the approved guidelines. The study was approved by the Hospital Universitario San Carlos Ethics Committee (Madrid), and all participants signed a written informed consent prior to participation.

### MEG Acquisition

Magnetoencephalography signals were measured by a 306 channel Vectorview system (Elekta Neuromag Oy) at the Center for Biomedical Technology (Madrid, Spain), inside a magnetically shielded room (VacuumSchmelze GmbH, Hanau, Germany) within several days after the neuropsychological assessment. Brain magnetic fields were recorded in the morning during a task-free 3 min eyes-closed resting-state condition, while subjects sat comfortably. Participants were instructed to move as little as possible, and were monitored during the recording to ensure that they did not fall asleep.

Sampling frequency was 1000 Hz with an online filter with bandwidth 0.1–300 Hz. The position of the head inside the sensor array was determined using a head-position indicator (HPI) with four coils attached to the scalp (two on the mastoids and two on the forehead). These four coils along with the head shape (∼500 points) of each subject (referenced to three anatomical fiducials: nasion, and left-right preauricular points) were acquired using a three-dimensional Fastrak Polhemus system (manufacturer: Polhemus, Inc., USA). Vertical ocular movements were measured by two bipolar electrodes attached above and below the left eye, and a third one to the earlobe, for electrical grounding.

Maxfilter software (version 2.2, Elekta Neuromag Oy) was used to remove noise from the MEG data using the temporal extension of signal space separation (tSSS) with movement compensation (Taulu and Simola, 2006). Flat channels, or those that contained excessive artifacts, were manually discarded after visual inspection of the data by one of the authors (M. E. López) before estimation of the SSS coefficients. The tSSS filter was subsequently used to remove noise signals that SSS failed to discard, typically from noise sources near the head, using a subspace correlation limit of 0.9 (Medvedovsky et al., 2009) and a sliding window of 10 s.

### MRI Acquisition

3D T1 weighted anatomical brain MRI scans were collected with a General Electric 1.5T MRI scanner, using a high resolution antenna and a homogenization PURE filter [Fast Spoiled Gradient Echo (FSPGR) sequence with parameters: TR/TE/TI = 11.2/4.2/450 ms; flip angle 12°; 1 mm slice thickness, a 256 × 256 matrix and FOV 25 cm].

FreeSurfer software (version 5.1.0; Fischl et al., 2002) was used to obtain the hippocampal volumes, which were normalized with the overall intracranial volume (ICV) of each subject.

### Co-registration and Beamforming

The outline of the scalp, as obtained from the subject’s structural MRI, was used for co-registration with the MEG data using the VUmc Amsterdam co-registration surface matching software, resulting in an estimated co-registration accuracy of approximately 4 mm (Whalen et al., 2008). A single sphere fitted to the scalp surface was used as a volume conductor model for the beamformer analysis. An atlas-based beamformer was used to project the MEG sensor signals to 78 cortical regions-of-interest (ROIs) from the Automatic Anatomical Labeling (AAL) atlas (see Supplementary Material) (Tzourio-Mazoyer et al., 2002; Gong et al., 2009). Based on the broad-band (0.5–48 Hz) beamformer weights, time series of neuronal activity were reconstructed for the voxel with the maximum power within a ROI for each frequency band separately, i.e., a virtual electrode that was representative for that specific ROI was reconstructed. A detailed description of this procedure is given in Hillebrand et al. (2012).

### MEG Analysis

Per subject, five artifact free trials of approximately 16.384 s (four times 4096 samples) were selected after careful visual inspection, giving a total of 20 epochs of 4096 samples for further analysis. Time-series of neuronal activation were computed for the six frequency bands: delta (0.5–4 Hz), theta (4–8 Hz), lower alpha (8–10 Hz), upper alpha (10–13 Hz), beta (13–30 Hz), and gamma (30–48 Hz). Selected epochs were converted to ASCII-files and imported into an in-house developed software package BrainWave version 0.9.125, developed by one of the authors (C. J. Stam) and available at: http://home.kpn.nl/stam7883/brainwave.html.

### Functional Connectivity

Functional connectivity was assessed with the PLI, which quantifies the consistency of a phase relationship between two signals while zero-lag (mod π) phase differences are ignored (Stam et al., 2007b). Therefore, the PLI is insensitive to spurious interactions caused by the effects of volume conduction and/or field spread (Stam et al., 2007b; Hillebrand et al., 2012). The PLI ranges between 0 and 1 in which 0 represents no consistent coupling or coupling with zero-lag and one represents consistent phase-lagged coupling. First, the instantaneous phase for each time series is computed by taking the argument of the analytic signal (Stam et al., 2007b) as computed using the Hilbert transform. Second, we calculate the asymmetry of the distribution of instantaneous phase differences between two time series:

$$\text{PLI = }|<\text{sign}\left[\text{sin}\left(\Delta \Phi_{\text{t}}\right)]>\right|$$

where the phase difference ΔΦ*_t_* is defined in the interval [-π, π], <> denotes the mean value, sign stands for signum function, || indicates the absolute value, and *t* corresponds to time samples 1, …, *N*s, where *N*s is the number of samples. By calculating the PLI values between all pairs of ROIs, we obtained a 78 × 78 adjacency matrix, which we used for the network analyses (see below).

### Small-Worldness

A low characteristic path length (L) and a high clustering coefficient (C) characterize a small-world network (Watts and Strogatz, 1998). In an unweighted network, the C represents the probability that two nodes are connected when they share a neighboring node and the L represents the average of the shortest distance between pairs of nodes, with distance defined by the number of links between nodes. From the weighted graph, the weighted clustering coefficient (Cw) and weighted characteristic path length (Lw) were calculated as described in Stam et al. (2007a). Fifty random control networks were created by randomly shuffling the PLI values in each adjacency matrix while keeping the matrix symmetry intact. For each ensemble of 50 random networks, the average Cw (random) and Lw (random) were computed. The observed network values were divided by the average values obtained for the random networks in order to create normalized values. The resulting normalized clustering coefficient (normalized Cw) and normalized path length (normalized Lw) were used for further analyses. These measures were computed for each epoch, and then averaged over the epochs for each subject.

### Minimum Spanning Tree

We constructed the MST from the weighted adjacency matrix containing the PLI values. The MST is a unique subgraph that connects all nodes in the network by the strongest connections (defined as the network links with the highest PLI values) without forming cycles (Stam et al., 2014), and was reconstructed using Kruskal’s algorithm (Kruskal, 1956). By using 1/PLI as input to the algorithm, strongest connections are likely to be included in the MST, as long as no cycles are formed. The MST was characterized by the following measures: degree, betweenness centrality (BC), eccentricity, degree distribution (κ), the number of leafs, degree correlation (R), tree hierarchy and diameter. MST measures were computed for each epoch, and then averaged over the epochs for each subject. The degree describes how many links each node has. BC is a measure of the importance of a node within the network. The BC of node *i* is defined as the number of shortest paths in the network that run through a specific node, divided by the total number of shortest paths from any node to all other nodes in the MST. In our calculations, we used the maximum BC across all nodes as well as per node for further testing. The eccentricity of a node is defined as the longest distance between that node and any other node in the network. The degree distribution is formed by the likelihood (P) that a randomly chosen node of the network will have degree κ (the number of connections of a specific node); it is a plot of P(κ) as a function of κ (Stam and Reijneveld, 2007). The degree correlation is an index of how much the degree of a node is correlated to the degree of nodes it is connected to. The leaf number is the number of nodes that have a degree of 1 representing the “leafs” or “extremities” of the network. Tree hierarchy quantifies the trade-off between large scale integration in the MST and the overload of central nodes. The diameter represents the longest path in the MST. For more information about these measures, we refer to Boersma et al. (2013), Stam et al. (2014), Tewarie et al. (2015).

### Statistical Analysis

**FIGURE 1:**
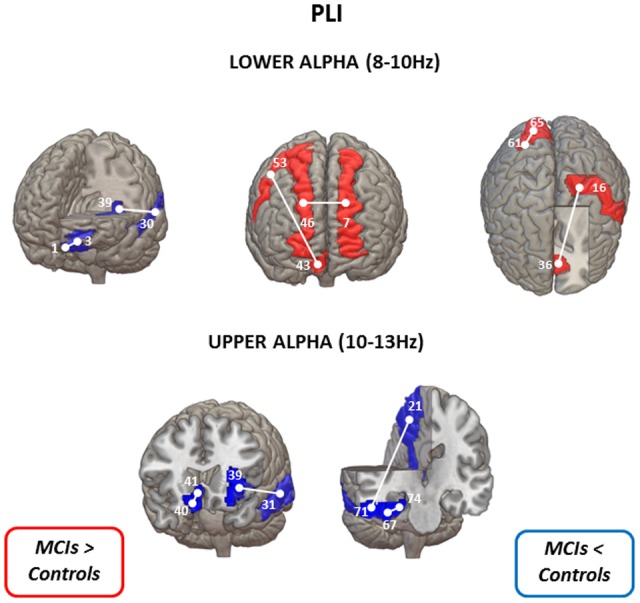
**Statistical differences between controls and MCIs in lower alpha **(upper panel)** and upper alpha **(lower panel)** bands in PLI values (uncorrected for multiple comparisons).** When MCI group show lower connectivity values than the control group is represented in blue: left gyrus rectus (1) – left superior frontal gyrus (3); left superior temporal gyrus (30) – left insula (39) for the lower alpha band; and left insula (39) – left middle temporal gyrus (31); right gyrus rectus (40) – right olfactory cortex (41); left precuneus (21) – right inferior temporal gyrus (71); right fusiform gyrus (67) – right parahippocampal gyrus for the upper alpha band. On the contrary, when MCI group exhibit higher PLI values than the control group is represented in red: left superior frontal gyrus (dorsolateral) (7) – right superior frontal gyrus (dorsolateral) (46); right superior frontal gyrus (medial orbital) (43) – right precentral gyrus (53) – left postcentral gyrus (16) – left anterior cingulate and paracingulate gyri (36) – right superior occipital gyrus (61) – right cuneus (65) for the lower alpha band. AAL numbers appear in parentheses.

**FIGURE 2:**
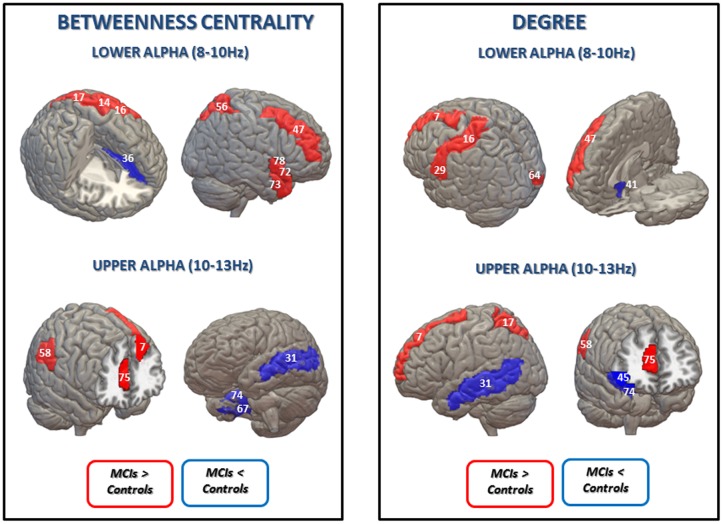
**Statistical differences between the control group and the MCI group in betweenness centrality (BC) **(Left)** and degree **(Right)** in lower and upper alpha bands (uncorrected for multiple comparisons).** Blue is represented when MCIs exhibit lower BC values than the control group in: left anterior cingulate and paracingulate gyri (36) for lower alpha band; left middle temporal gyrus (31), right fusiform gyrus (67) and right parahippocampal gyrus (74) for the upper alpha band; and in degree values in: right olfactory cortex (41) for the lower alpha band; left middle temporal gyrus (31), right inferior frontal gyrus, orbital part (45) and right parahippocampal gyrus (74) for the upper alpha band. Red is represented when the MCI present higher BC values in: left precentral gyrus (14), left postcentral gyrus (16); left superior parietal gyrus (17), right middle frontal gyrus (47), right superior parietal gyrus (56), right temporal pole: superior temporal gyrus (72), right temporal pole: middle temporal gyrus (73) and right Insula for the lower alpha band; left superior frontal gyrus, dorsolateral (7), right supramarginal gyrus (58), and right anterior cingulate and paracingulate gyri (75) for the upper alpha band; and higher degree values in: left superior frontal gyrus, dorsolateral (7), left postcentral gyrus (16), left Heschl gyrus (29) and right middle frontal gyrus (47) for the lower alpha band; left superior frontal gyrus, dorsolateral (7), left superior parietal gyrus (17), right supramarginal gyrus (58) and right anterior cingulate and paracingulate gyri (75) for the upper alpha band. AAL numbers appear in parentheses.

Subject’s characteristics were tested using independent samples *t*-tests or chi-square tests where appropriate using SPSS (20.0 for windows). For the PLI, permutation testing, based upon *t*-statistics, was used for each pair of regions [among the 78 studied, (78 × 77)/2 in total], and in each frequency band, with the aim of comparing both groups (Maris and Oostenveld, 2007). To this end, participants were randomly divided into two sets with the same size as the original groups (29 vs. 29 subjects). This procedure was repeated 2000 times (2000 permutations). A new *t*-test between each pair of regions [(78 × 77)/2 couples] in each frequency band, was then carried out using these two newly created groups, getting a *t*-value for each pair of regions and each frequency band. After sorting these 2001 *t*-test results [2000 corresponding to “randomly divided” groups and another one for the original (MCI vs. Control) subject’s distribution], only *p*-values within the 5% of lower values were considered statistically significant [note that this is for each pair of regions and each frequency band studied: hence it was repeated ((78 × 77)/2) × (number of frequency bands) times]. These analyses were uncorrected for the number of permutations performed and therefore serve as exploratory analyses (see **Figures 1**, **2** and **Tables 2**, **3**). Afterward, we performed an FDR correction on the data with the goal to examine the statistical significant results that survive a multiple comparison correction. For the network analysis, we focused on those frequency bands in which the connectivity analyses showed (uncorrected) significant differences between the groups. Again, corrected and uncorrected (exploratory) permutation testing was used to compare the groups. All statistical analyses were performed using MATLAB (R2015b, Mathworks).

## Results

### Demographics

Subject characteristics are shown in **Table 1**. Controls and MCI patients did not differ in age, gender, or educational level. As expected, the scores of MMSE and two measures of episodic memory (immediate and delayed recall) were both lower in MCI patients compared to controls. Additionally, hippocampal volumes were both lower in the MCI group.

**Table 1:** Subject’s characteristics (Control group, *n* = 29; MCI group, *n* = 29).

	Group	Mean ± SD	*p*-values
Age (years)	Control	70.62 ± 3.913	0.766^∗^
	MCI	72.55 ± 4.163
Gender (M/F)	Control	10/19 ± 0.484	0.096^∗∗^
	MCI	15/14 ± 0.509
Educational level	Control	3.72 ± 1.162	0.202^∗^
	MCI	2.68 ± 1.362
MMSE	Control	29.38 ± 0.68	<0.001^∗^
	MCI	27.21 ± 1.75
Immediate recall	Control	39.14 ± 8.05	<0.001^∗^
	MCI	18.11 ± 10.03
Delayed recall	Control	24.83 ± 7.18	<0.001^∗^
	MCI	7.59 ± 8.35
RH_ICV	Control	0.0026 ± 0.0003	0.004^∗^
	MCI	0.0021 ± 0.0006
LH_ICV	Control	0.0025 ± 0.0003	0.006^∗^
	MCI	0.0022 ± 0.0005


*Mean ± standard deviation (SD) and *p*-values. The *p*-values were obtained by two-independent samples *t*-test (^∗^) or chi-square test (^∗∗^) where relevant. M, male; F, female; MMSE, Mini Mental State Examination score; RH_ICV and LH_ICV, right and left hippocampal volume normalized with intracranial volume, respectively. Highest education completed, using five levels: (1) Illiterate, (2) Primary studies, (3) Elemental studies, (4) High school studies, and (5) University studies.*

### Functional Connectivity

Significant differences between MCI patients and controls in PLI using the uncorrected permutation tests were obtained in the lower (8–10 Hz) and upper (10–13 Hz) alpha band (**Figure 1**). In the lower alpha band, compared to the control group, PLI in the MCI group was lower between left superior frontal gyrus -orbital part- and left gyrus rectus, and between left superior temporal gyrus and left insula (*p* < 0.01). Additionally, in the same frequency band, MCI subjects showed higher PLI values than controls between four regions, namely between right superior frontal gyrus (medial orbital) and right precentral gyrus, right superior frontal gyrus (dorsolateral) and left superior frontal gyrus (dorsolateral), left anterior cingulate and paracingulate gyri and left postcentral gyrus, and between right cuneus and right superior occipital gyrus.

Furthermore, in the upper alpha band, MCI subjects presented lower PLI values than controls between four regions, namely between right gyrus rectus and right olfactory cortex, left insula and left middle temporal gyrus, right parahippocampal gyrus and right fusiform gyrus, and right inferior temporal gyrus and left precuneus.

After FDR-correction for multiple comparisons, none of these significant differences survived.

### Small-worldness

We focused the network analysis on those frequency bands in which there were differences in connectivity, namely in lower and upper alpha bands.

Using the uncorrected permutation tests, no differences between controls and MCI subjects were found for normalized Cw and normalized Lw.

### Minimum Spanning Tree

We found group differences in BC and degree in lower and upper alpha bands using the uncorrected permutation tests (see below).

Betweenness centrality results are shown in **Figure 2** and **Table 2**. In the lower alpha band, there were no differences between controls and MCI patients in the maximum value of BC globally. However, compared to the healthy controls, the MCI group showed higher BC values in eight brain areas: the left pre- and post-central gyri, the left and right superior parietal gyri, the right middle frontal gyrus, the right superior and middle temporal gyrus (temporal pole) and the right insula; and lower values in one brain area: the left anterior cingulated/paracingulate gyri (see Supplementary Material).

**Table 2:** Mean ± standard deviation (SD) of the betweenness centrality (BC) in the lower and upper alpha band, *p*-values were obtained using permutation testing without correction for multiple comparisons across regions.

MST BC				
**Name of the area**	**Group**	**Mean ± *SD***	***p*-values**	**Change**

**Lower alpha band**				
Left precentral gyrus (lPreCG)	Control	0.0454 ± 0.0039	0.034	↑MCI
	MCI	0.0703 ± 0.0050
Left postcentral gyrus (lPoCG)	Control	0.0363 ± 0.0016	0.035	↑MCI
	MCI	0.0636 ± 0.0035
Left superior parietal gyrus (lSPG)	Control	0.0629 ± 0.0043	0.037	↑MCI
	MCI	0.0984 ± 0.0050
Right middle frontal gyrus (rMFG)	Control	0.0266 ± 0.0013	0.041	↑MCI
	MCI	0.0595 ± 0.0043
Right superior parietal gyrus (rSPG)	Control	0.2978 ± 0.0097	0.012	↑MCI
	MCI	0.3627 ± 0.0068
Right temporal pole: superior temporal gyrus (rTPOsup)	Control	0.2673 ± 0.0032	0.012	↑MCI
	MCI	0.3118 ± 0.0041
Right temporal pole: middle temporal gyrus (rTPOmid)	Control	0.2561 ± 0.0027	0.003	↑MCI
	MCI	0.3019 ± 0.0036
Right insula (rINS)	Control	0.2644 ± 0.0020	0.032	↑MCI
	MCI	0.3079 ± 0.0067
Left anterior cingulate and paracingulate gyri (lACG)	Control	0.1077 ± 0.0037	0.002	↓MCI
	MCI	0.0629 ± 0.0034
**Upper alpha band**				
Left superior frontal gyrus, dorsolateral (lSFGdor)	Control	0.0443 ± 0.0034	0.018	↑MCI
	MCI	0.0897 ± 0.0079
Right supramarginal gyrus (rSMG)	Control	0.0593 ± 0.0055	0.012	↑MCI
	MCI	0.0967 ± 0.0059
Right anterior cingulate and paracingulate gyri (rACG)	Control	0.0320 ± 0.0012	0.014	↑MCI
	MCI	0.0722 ± 0.0040
Left middle temporal gyrus (lMTG)	Control	0.0998 ± 0.0096	0.014	↓MCI
	MCI	0.0414 ± 0.0017
Right fusiform gyrus (rFFG)	Control	0.0824 ± 0.0032	0.048	↓MCI
	MCI	0.0586 ± 0.0035
Right parahippocampal gyrus (rPHG)	Control	0.0904 ± 0.0044	0.004	↓MCI
	MCI	0.0539 ± 0.0057


*Arrows indicate lower or higher values in the MCI group.*

In the upper alpha band, we did not find global differences between the groups, but there were differences in BC for specific brain areas. Compared to controls, MCIs exhibited higher values in three brain areas: the left superior frontal gyrus dorsolateral (lSFGdor), the right supramarginal gyrus (rSMG), and the right anterior cingulate/paracingulate gyri (rACG); and lower BC values in three brain areas: the left middle temporal gyrus (lMTG), the right fusiform gyrus (rFFG) and the right parahippocampal gyrus (rPHG).

Finally, the MCI group showed higher MST degree values than controls in the lower alpha band in five brain areas: the left superior frontal gyrus, dorsolateral, the left postcentral gyrus, the left Heschl gyrus, the right middle frontal gyrus and the right calcarine fissure and surrounding cortex; while they exhibited a lower degree value in one brain area: the right olfactory cortex. In the upper alpha band, MCI subjects exhibited higher degree values in four brain areas: the left superior frontal gyrus, dorsolateral, the left superior parietal gyrus, the rSMG and the right anterior cingulate and paracingulate gyri; and lower degree values in three brain areas: the lMTG, the right inferior frontal gyrus, orbital part and the rPHG. MST degree results are shown in **Figure 2** and **Table 3**.

**Table 3:** Mean ± standard deviation (SD) and *p*-values after a *t*-test without correction for multiple comparisons for degree values in the control group compared with the MCI group in lower and upper alpha bands.

MST DEGREE				
**Name of the area**	**Group**	**Mean ± *SD***	***p*-values**	**Change**

**Lower alpha band**				
Left superior frontal gyrus, dorsolateral (lSFGdor)	Control	0.0221 ± 8.3 × 10^-5^	0.007	↑MCI
	MCI	0.0272 ± 8.5 × 10^-5^
Left postcentral gyrus (lPoCG)	Control	0.0204 ± 3.7 × 10^-5^	0.010	↑MCI
	MCI	0.0247 ± 4.8 × 10^-5^
Left Heschl gyrus (lHES)	Control	0.0244 ± 8.2 × 10^-5^	0.038	↑MCI
	MCI	0.0302 ± 1.2 × 10^-4^
Right middle frontal gyrus (rMFG)	Control	0.0186 ± 3.3 × 10^-5^	0.008	↑MCI
	MCI	0.0238 ± 7.1 × 10^-5^
Right calcarine fissure and surrounding cortex (rCAL)	Control	0.2559 ± 0.0041	0.022	↑MCI
	MCI	0.2966 ± 0.0052
Right olfactory cortex (rOLF)	Control	0.0280 ± 8.2 × 10^-5^	0.044	↓MCI
	MCI	0.0230 ± 5.3 × 10^-5^
**Upper alpha band**				
Left superior frontal gyrus, dorsolateral (lSFGdor)	Control	0.0224 ± 9.9 × 10^-5^	0.035	↑MCI
	MCI	0.0278 ± 1.3 × 10^-4^
Left superior parietal gyrus (lSPG)	Control	0.0242 ± 9.2 × 10^-5^	0.021	↑MCI
	MCI	0.0320 ± 2.2 × 10^-4^
Right supramarginal gyrus (rSMG)	Control	0.0243 ± 1.0 × 10^-4^	0.049	↑MCI
	MCI	0.0291 ± 9.7 × 10^-5^
Right anterior cingulate and paracingulate gyri (rACG)	Control	0.0196 ± 1.2 × 10^-5^	0.043	↑MCI
	MCI	0.0241 ± 7.5 × 10^-5^
Left middle temporal gyrus (lMTG)	Control	0.0297 ± 2 × 10^-4^	0.030	↓MCI
	MCI	0.0220 ± 5.6 × 10^-5^
Right inferior frontal gyrus, orbital part (rORBinf)	Control	0.0266 ± 8.5 × 10^-5^	0.023	↓MCI
	MCI	0.0213 ± 1.7 × 10^-5^
Right parahippocampal gyrus (rPHG)	Control	0.0255 ± 6.5 × 10^-5^	0.008	↓MCI
	MCI	0.0211 ± 9 × 10^-5^


*Arrows indicate lower and higher values in the MCI group.*

After FDR-correction for multiple comparisons, none of these significant differences survived. Also, we did not find any differences between the controls and the MCI group for any of the other MST measures in these two frequency bands.

## Discussion

With the aim to corroborate our hypothesis about the differences in both functional connectivity and network organization between healthy aging and MCI, we performed a functional connectivity (PLI) and MST analyses in resting state MEG data. The main finding of this study was the detection of differences in both functional connectivity and brain network topology in a group of patients with MCI compared to controls. Note however, that these results are exploratory and the significance between the groups did not survive FDR correction for multiple comparisons. The uncorrected connectivity results showed that the MCI patients exhibited more increases than decreases in PLI values in the lower alpha band, and decreases in the upper alpha band. As differences in connectivity between both groups were found in the alpha band, we examined differences of network’s topography in this frequency band by using concepts from graph theory. We did not find any group difference in weighted clustering and path length, but regionally we obtained higher BC and degree values when examining the MST in the MCI group in lower alpha band, and both increases and decreases in the upper alpha band.

Mild cognitive impairment patients demonstrated lower PLI values in the lower alpha band that affected frontal and temporal brain areas within the left hemisphere. Using EEG, an overall decrease in the lower alpha band has been observed in AD patients (Stam et al., 2006, 2009), and also in MCI patients (Babiloni et al., 2006). In a recent study performed with EEG data in AD (Engels et al., 2015), the decrease of connectivity in the lower alpha band was related to the severity of the disease, mainly over posterior areas. However, in the present study, MCI patients also showed an increase in connectivity between intra- and inter-hemispheric frontal areas, and in right posterior regions. This intra- and inter-hemispheric increase in connectivity has been usually described in the MCI population while performing a cognitive task. Pijnenburg et al. (2004) found an increase in lower alpha band in MCIs compared to subjects with subjective memory complaints (SMC) during a visual working memory (WM) task. Jiang (2005) and Zheng et al. (2007) obtained higher coherence values in both lower and upper alpha bands during an arithmetic WM paradigm in MCIs compared to healthy controls. In addition, an MEG study performed in progressive MCI patients (pMCI) found a higher synchronization in those patients who finally developed AD, compared with those who remained stable over time (stable MCI, sMCI), in lower alpha and upper alpha bands while performing a memory task (Bajo et al., 2012). In the same vein, a recent resting-state MEG study which did not divide the alpha band into two sub-bands, found that patients with MCI that eventually converted to AD, exhibited a higher connectivity in this frequency range than those MCI patients that remained stable over time, between the right anterior cingulate and temporo-occipital brain regions (López et al., 2014a). Our findings add to the current knowledge that results of functional connectivity in MCI patients are dependent on the region and on the frequency band. However, there is no consistent increase or decrease in connectivity in patients with MCI compared to controls during resting state. Therefore, we conclude that, the increases and decreases of functional connections observed in the MCI population in the lower alpha band may reflect the aberrant functioning until the breakdown of the system, which characterizes AD.

The increase in PLI values found in the lower alpha band in patients with MCI has been commonly considered as a compensatory mechanism. This interpretation was related to the attempt of the brain to overcome the damage caused by the disease in the networks involved in cognitive functioning (see Grady, 2012; Scheller et al., 2014 for reviews). In the case of healthy controls, this mechanism would not be needed while AD patients would not compensate any more due to the severity of the disease. Nonetheless, recent studies postulated that instead of being a compensatory mechanism, it would be a pathological characteristic of MCI patients (de Haan et al., 2012; López et al., 2014a,b). During the course of the disease, there is a loss of GABAergic synapsis caused by the accumulation of β-amyloid (Aβ) plaques (Garcia-Marin et al., 2009), producing an inhibitory deficit. The loss of inhibitory interneurons in the cortex would induce increasing brain activity/connectivity in MCI patients, leading to an aberrant functioning (disinhibition) until the breakdown of the system, which is what occurs in AD.

In agreement with what has previously been described in some AD studies (Stam et al., 2002; Pijnenburg et al., 2004), we obtained lower connectivity values in the MCI group in the upper alpha band mainly concerning temporal and parietal brain areas. As far as we know, no studies have been performed describing this finding in MCI. However, considering the alpha band as one (normally from 8 to 13 Hz), some authors have revealed this decrease in connectivity in MCI patients compared to controls (Koenig et al., 2005; Garcés et al., 2014; Cuesta et al., 2015). Our results point out that networks that are usually implicated in episodic memory, olfactory function, visuospatial processing or executive functioning (previously described in the Results section) are already impaired in MCI patients. These results may indicate that in MCI the disconnection that characterizes AD would have already started, probably contributing to the cognitive deficits observed in this population. According to the increases and decreases obtained in PLI values, which have been also described in previous studies, it might be considered that during the symptomatic pre-dementia phase of AD, two mechanisms could be coexisting in MCI: disconnection and aberrant functioning.

To elucidate about the meaning of this duality of hyper and hypo connectivity we decided to evaluate the functional network organization using the network theory approach. We started with two of the most basic network parameters: the characteristic path length and the clustering coefficient. As firstly described by Watts and Strogatz (1998), these two measures together form the concept of the small-world network topology whereas the network architecture combines an efficient balance between local (short range) and global (long range) connectivity. This small-world configuration is thought to be better suited for information transfer and thus presumably for cognitive processing rather than the topology of random or regular networks (Bassett and Bullmore, 2006; Stam et al., 2007a). We did not find differences in terms of clustering and path length in our MCI cohort. In other studies, however, an increased path length and decreased clustering coefficient in MCI was found (Xiang et al., 2013; Zhang et al., 2015) and therefore MCI mimics results of AD studies (Stam et al., 2007a). MCI has been referred to as an intermediate state between healthy aging and AD in terms of their network topology (Seo et al., 2013). Our cohort did not differ in terms of the small-world parameters clustering coefficient and characteristic path length and therefore the exploration of different network measures is interesting since they may be more sensitive for the subtle changes in MCI.

Studying brain networks using measures like the clustering coefficient and the characteristic path length give useful insights within datasets of similar network sizes and link densities, but cause a comparison problem when these requirements are not met. This problem is thoroughly explained in a paper by van Wijk et al. (2010). It stresses the comparison problem between networks, not only because of the differences in network sizes (number of nodes) and degree but also due to arbitrary choices that have to be made (i.e., the threshold for the link density within a weighted network). This was the main reason for the use of the MST (Stam, 2014). Using MST, no arbitrary choices have to be made in case of unique functional connectivity values: it does not require setting a threshold and the number of nodes and links is fixed. It can be regarded as the backbone of a network (Çiftçi, 2011; Yu et al., 2015). In the present study, we found differences in two measures of centrality when comparing the MST of MCI patients and healthy controls. The MST is regarded as the backbone of a functional network since it merely involves the strongest links of the network (Stam, 2014). The MST-BC as a measure for centrality has previously shown a shifted hub location in patients with AD in high frequency bands (Engels et al., 2015). In our study, we found increased BC values in MCI patients as well as some decreases in lower and upper alpha bands. The degree, also a measure of centrality, was also found to be reduced mainly in the temporal regions. As with the functional connectivity measures, we thus found a dual pattern in the MCI population. These findings may suggest that the loss of BC/degree, mainly in temporal areas, may reflect that these areas are weakened in the brain network while frontal and parietal compensate for this malfunction. It also may reflect that some brain areas lose control within the network while others are functioning in a more aberrant way. In conclusion, although after correcting for multiple comparisons the significant differences in MST disappeared, this study showed that the classical network measures (normalized Lw and Cw) did not distinguish between MCIs and controls during resting state, but MST analysis may be a new and useful procedure to characterize and differentiate both populations. Although the reduction in centrality in temporal regions was not reported in the one study evaluating the MST BC in AD (Engels et al., 2015), this finding can be understood in the light of the disease pathology, which involves the temporal lobe. The differences between these two studies may be explained by differences in age difference (patients in our cohort were older) and therefore parietal pathology may be relatively less present (Adriaanse et al., 2012).

This study has a number of strengths and limitations. A strong point is that we used the PLI as a measure of functional connectivity since it reduces the bias due to volume conduction and/or field spread (Stam et al., 2007b). Another strong point is the use of conventional network measures (i.e., the normalized clustering coefficient and the characteristic path length), which are well described in literature, and MST parameters, that offer an arbitrary-free method for comparing networks with different properties. Our source-space analyses included 78 regions of interest according to the AAL atlas. This is a commonly used atlas, but our approach could be applied to other atlases as well. Besides these advantages, this study has several limitations as well which should be taken into account. Our results may have been influenced by methodological choices such as the selection of artifact-free epochs by one of the authors (M. M. Engels). Epochs with signs of artifacts, drowsiness were discarded. One of the other authors (M. E. López) checked the selected epochs and therefore, we expect that the epochs we have selected for our final analyses are artifact-free. An important consideration for this approach was that we did not want to apply data cleaning approaches (e.g., Delorme et al., 2006) that could modify the connectivity structure of the data, and thereby bias subsequent functional connectivity and network analyses. Consistent with our previous work, we therefore opted to rely on thorough visual inspection for the selection of artifact free data segments.

Finally, it should be pointed that our MCIs were recruited from a clinical context. Several studies have reported that it is easier to find more cases of MCIs within a clinical population and also that the rate of conversion to AD per year is higher in a clinical setting compared to the general population (Farias et al., 2005; Jelic et al., 2006). Although NIA-AA clinical criteria is standard for all subjects (Albert et al., 2011), our findings may be more representative of the clinical than the community population.

Please note that the significant differences described in this study were not corrected for multiple testing. The FDR-corrected results did not show any significant group differences. Therefore, these results are presented as an exploratory study that can be used as a guide for regions and measures that show a trend toward significance between MCI and controls.

Our results revealed differences between MCI patients and controls. These patients did not have dementia yet, although they have an increased risk of developing it. Although these patients only have minor cognitive deficits, the functional connectivity and network differences are striking, suggesting a possible causative role. Therefore, measures of functional connectivity, and the network parameters derived from these inter-areal functional connections, may help to characterize the very early stages of dementia.

## Author Contributions

ML performed the MEG recordings, wrote the main manuscript, and prepared the figures; ME pre-processed the MEG data, collaborated with the MEG data analysis, and wrote the main manuscript; EvS supervised the study; RB collaborated with the statistical analysis; MD collected the sample; PS supervised the study; AH collaborated with the statistical analysis and supervised the study; CS supervised the study; FM collaborated with the experimental design and supervised the study. All authors reviewed the manuscript.

## Conflict of Interest Statement

The authors declare that the research was conducted in the absence of any commercial or financial relationships that could be construed as a potential conflict of interest.
