# Supplementary material for: MEG Beamformer-Based Reconstructions of Functional Networks in Mild Cognitive Impairment
Source: Front Aging Neurosci. 2017 Apr 25;9:107. doi: 10.3389/fnagi.2017.00107 (PMC5403893; doi:10.3389/fnagi.2017.00107)
Supplement: Supplementary file 1 [file Table_1.docx]

Supplementary Material

*“MEG beamformer-based reconstructions of functional networks in mild cognitive impairment”*

M.E. López^*^, M.M.A. Engels^*^, E.C.W. van Straaten, R. Bajo, M.L. Delgado, Ph.Scheltens, A. Hillebrand, C.J. Stam, F. Maestú

Correspondence: [meugenia.lopez@uib.es](mailto:meugenia.lopez@uib.es)

***Brain areas from the Automatic Anatomical Labeling (AAL) atlas***

| **AAL number** | **Hemisphere** | **Cortical regions** | **Abbreviations** |
| --- | --- | --- | --- |
| 1 | Left | Gyrus Rectus | REC |
| 2 | Left | Olfactory Cortex | OLF |
| 3 | Left | Superior frontal gyrus, orbital part | ORBsup |
| 4 | Left | Superior frontal gyrus, medial orbital | ORBsupmed |
| 5 | Left | Middle frontal gyrus orbital part | ORBmid |
| 6 | Left | Inferior frontal gyrus, orbital part | ORBinf |
| 7 | Left | Superior frontal gyrus, dorsolateral | SFGdor |
| 8 | Left | Middle frontal gyrus | MFG |
| 9 | Left | Inferior frontal gyrus, opercular part | IFGoperc |
| 10 | Left | Inferior frontal gyrus, triangular part | IFGtriang |
| 11 | Left | Superior frontal gyrus, medial | SFGmed |
| 12 | Left | Supplementary motor area | SMA |
| 13 | Left | Paracentral lobule | PCL |
| 14 | Left | Precentral gyrus | PreCG |
| 15 | Left | Rolandic operculum | ROL |
| 16 | Left | Postcentral gyrus | PoCG |
| 17 | Left | Superior parietal gyrus | SPG |
| 18 | Left | Inferior parietal, but supramarginal and angular gyri | IPL |
| 19 | Left | Supramarginal gyrus | SMG |
| 20 | Left | Angular gyrus | ANG |
| 21 | Left | Precuneus | PCUN |
| 22 | Left | Superior occipital gyrus | SOG |
| 23 | Left | Middle occipital gyrus | MOG |
| 24 | Left | Inferior occipital gyrus | IOG |
| 25 | Left | Calcarine fissure and surrounding cortex | CAL |
| 26 | Left | Cuneus | CUN |
| 27 | Left | Lingual gyrus | LING |
| 28 | Left | Fusiform gyrus | FFG |
| 29 | Left | Heschl gyrus | HES |
| 30 | Left | Superior temporal gyrus | STG |
| 31 | Left | Middle temporal gyrus | MTG |
| 32 | Left | Inferior temporal gyrus | ITG |
| 33 | Left | Temporal pole: superior temporal gyrus | TPOsup |
| 34 | Left | Temporal pole: middle temporal gyrus | TPOmid |
| 35 | Left | Parahippocampal gyrus | PHG |
| 36 | Left | Anterior cingulate and paracingulate gyri | ACG |
| 37 | Left | Median cingulate and paracingulate gyri | DCG |
| 38 | Left | Posterior cingulate gyrus | PCG |
| 39 | Left | Insula | INS |
| 40 | Right | Gyrus Rectus | REC |
| 41 | Right | Olfactory Cortex | OLF |
| 42 | Right | Superior frontal gyrus, orbital part | ORBsup |
| 43 | Right | Superior frontal gyrus, medial orbital | ORBsupmed |
| 44 | Right | Middle frontal gyrus orbital part | ORBmid |
| 45 | Right | Inferior frontal gyrus, orbital part | ORBinf |
| 46 | Right | Superior frontal gyrus, dorsolateral | SFGdor |
| 47 | Right | Middle frontal gyrus | MFG |
| 48 | Right | Inferior frontal gyrus, opercular part | IFGoperc |
| 49 | Right | Inferior frontal gyrus, triangular part | IFGtriang |
| 50 | Right | Superior frontal gyrus, medial | SFGmed |
| 51 | Right | Supplementary motor area | SMA |
| 52 | Right | Paracentral lobule | PCL |
| 53 | Right | Precentral gyrus | PreCG |
| 54 | Right | Rolandic operculum | ROL |
| 55 | Right | Postcentral gyrus | PoCG |
| 56 | Right | Superior parietal gyrus | SPG |
| 57 | Right | Inferior parietal, but supramarginal and angular gyri | IPL |
| 58 | Right | Supramarginal gyrus | SMG |
| 59 | Right | Angular gyrus | ANG |
| 60 | Right | Precuneus | PCUN |
| 61 | Right | Superior occipital gyrus | SOG |
| 62 | Right | Middle occipital gyrus | MOG |
| 63 | Right | Inferior occipital gyrus | IOG |
| 64 | Right | Calcarine fissure and surrounding cortex | CAL |
| 65 | Right | Cuneus | CUN |
| 66 | Right | Lingual gyrus | LING |
| 67 | Right | Fusiform gyrus | FFG |
| 68 | Right | Heschl gyrus | HES |
| 69 | Right | Superior temporal gyrus | STG |
| 70 | Right | Middle temporal gyrus | MTG |
| 71 | Right | Inferior temporal gyrus | ITG |
| 72 | Right | Temporal pole: superior temporal gyrus | TPOsup |
| 73 | Right | Temporal pole: middle temporal gyrus | TPOmid |
| 74 | Right | Parahippocampal gyrus | PHG |
| 75 | Right | Anterior cingulate and paracingulate gyri | ACG |
| 76 | Right | Median cingulate and paracingulate gyri | DCG |
| 77 | Right | Posterior cingulate gyrus | PCG |
| 78 | Right | Insula | INS |
